# Examining a possible association between human papilloma virus (HPV) vaccination and migraine: results of a cohort study in the Netherlands

**DOI:** 10.1007/s00431-014-2444-x

**Published:** 2014-11-01

**Authors:** T. M. Schurink-van’t Klooster, M. A. J. de Ridder, J. M. Kemmeren, J. van der Lei, F. Dekker, M. Sturkenboom, H. E. de Melker

**Affiliations:** 1 Department National Immunisation Programme, Centre for Infectious Disease Control, National Institute for Public Health and the Environment, PO box 1, 3720 BA Bilthoven, The Netherlands; 2 Department of Medical Informatics, Erasmus Medical Centre Rotterdam, Rotterdam, The Netherlands; 3 Public Health and Primary Care, Leiden University Medical Centre, Leiden, The Netherlands

**Keywords:** HPV, Human papilloma virus, Bivalent vaccine, Safety, Migraine

## Abstract

Since the introduction of the bivalent human papilloma virus (HPV) vaccine in the Netherlands, migraine has been reported as a notable event in the passive safety surveillance system. Research on the association between HPV vaccination and migraine is needed. Therefore, potential migraine cases in 2008–2010 were selected from a group of general practitioners and linked to the vaccination registry. Data were analysed in three ways: (i) incidences of migraine postvaccination (2009/2010) were compared to pre-vaccination incidences (2008); (ii) in a cohort, incidence rates of migraine in vaccinated and unvaccinated girls were compared and (iii) in a self-controlled case series analysis, the relative incidence of migraine in potentially high-risk periods was compared to non-high-risk periods. Incidence rates of migraine for 12- to 16-year-old girls and boys postvaccination were slightly higher than pre-vaccination incidence rates. Incidence rate ratios (IRRs) for vaccinated compared to unvaccinated girls were not statistically significantly higher. Furthermore, the RR for migraine in the high-risk period of 6 weeks following each dose versus non-high-risk period was 4.3 (95% confidence interval (CI) 0.69–26.6) for certain migraine.

*Conclusion*: Using different methods, no statistically significant association between HPV vaccination and incident migraine was found. However, the number of cases was low; to definitively exclude the risk, an increased sample size is needed.

## Introduction

Vaccination against human papilloma virus (HPV) with the bivalent HPV-16/18 vaccine (Cervarix®) was introduced in the Dutch National Immunisation Programme (NIP) in 2010 and has been provided annually to 12-year-old girls born in 1997 or later. Prior to the introduction of the vaccination into the NIP, a catch-up campaign was organised in 2009 for 13- to 16-year-old girls (i.e. born in 1993–1996) [8]. This campaign was extended in 2010 for girls who were not completely vaccinated. Vaccination coverage in 2009 and 2010 ranged per birth cohort between 49 and 56 %.

In the Netherlands, a national enhanced passive safety surveillance system has been in place since 1962. Reporting criteria are broad, and adverse events following immunisation can be reported by telephone or digitally. A new and notable event in the enhanced passive safety surveillance system for adverse events since the introduction of HPV vaccination was the number of reports of migraine. In 2009 and 2010, 52 reports of headache have been received following almost 800,000 doses of HPV vaccine [30]. Eight cases were diagnosed as migraines, of which three of the patients were born in 1994, one in 1995, two in 1996 and two in 1997. Headaches are known to be frequently reported following administration of the HPV vaccine [9, 11, 19]. However, migraine headaches can be severe, and attacks can occur regularly. Because background incidence rates for migraines are lacking, it is unknown whether the number of cases is more frequent in comparison to what can be expected for girls in this age range.

For seven out of the eight reports of migraine in the passive safety surveillance system, an expert panel assessed that it was improbable that the cause was vaccination [25, 26]. This assessment was based on the following points of consideration: diagnosis with severity and duration, time interval, biological plausibility, specificity of the symptoms, indications for other causes, proof for vaccine causation and underlying illness or concomitant health problems. In addition, no plausible pathophysiological mechanism is known to explain how migraine may be caused by HPV vaccination. Vaccination may act as a trigger for migraine, i.e. provocative factor for an attack. Although, according to many experts, the value of triggers is overestimated [14].

Migraine is characterised by recurrent moderate to severe attacks of usually unilateral, pulsating headaches with nausea and/or vomiting. The headache worsens with physical activity, and there is often increased sensitivity to light (photophobia) and sounds (phonophobia). Headaches last from 4 to 72 h in patients 15 years and older [13] and from 1 to 72 h in children younger than 15 years old [3, 15]. Migraine may be preceded by an aura, a transient focal neurological phenomenon that usually is visual [13]. The cause of migraine is unknown, and the value of triggers that cause an attack is difficult to prove. Migraines can occur in any patient from approximately 3 years of age. In childhood, migraine is more common in boys than in girls, but in puberty, the incidence of migraine in girls rises above that in boys [2]. The duration of migraine attacks in children is different than attacks in adults; the duration can be shorter in children and often lasts for only 1 h [3]. Therefore, doctors do not always identify migraines in children [12] and often misdiagnose migraines as tension headaches. In puberty, the character of the attacks changes, leading to a diagnosis of migraine.

The estimated lifetime prevalence of migraine for 20- to 65-year-old Dutch women is 33 % [18]. The prevalence of migraine in children 6–16 years old, with a case definition of headache attacks lasting 2–8 h associated with symptoms such as photophobia or nausea or preceded by an aura, is estimated at 8 % [28]. The incidence in general practices is approximately one new migraine patient per month [17].

In a previous study in the Dutch general population [32], we analysed the long-term occurrence of headache incidence in HPV-vaccinated and unvaccinated girls more than 1 year after the HPV vaccination campaign. At 14 years of age, no higher risk of having headache attacks (odds ratio (OR) 0.67, 95 % confidence interval (CI) 0.37–1.21), self-reported migraine or severe headaches (OR 0.96, 95 % CI 0.40–2.34) and migraine symptoms (i.e. headache attacks mostly during 1 h or more and at least two of the following characteristics: a pulsating pain, unilateral, severe enough to avoid going to school or other activities; OR 0.39, 95 % CI 0.15–1.00), with a first attack on or after 12 years of age, was found in the 12 months before the study questionnaire administration for HPV-vaccinated girls (*n* = 751) compared with unvaccinated girls (*n* = 368) [27]. Moreover, in this study, we aimed to investigate whether there is an association between HPV vaccination and incident migraine on a short term following administration of the bivalent HPV vaccine by using three different study designs. Even in a small number, reports of adverse events can easily lead to negative attention towards HPV vaccination. Therefore, research on the association between HPV vaccination and migraine is necessary to maintain trust in the NIP.

## Materials and methods

### Design

First, we applied a population-based study to estimate the incidence of migraine in the population pre- and post-introduction of the HPV vaccination programme. Furthermore, we conducted a cohort study and a self-controlled case series (SCCS) analysis in girls from 20 general practitioners (GPs) who agreed to provide data for linkage with the vaccination registry to assess the association between HPV vaccination and migraine.

### Setting

The department of Medical Informatics of the Erasmus University Rotterdam developed an electronic database of medical records from Dutch GPs, the Integrated Primary Care Information (IPCI) database. The database is designed for post-marketing surveillance and pharmaco-epidemiological research [31]. IPCI is a longitudinal observational database, which contains digital medical patient records from GPs in the Netherlands. At present, the database contains demographic information, medical notes, prescriptions and indications for therapy, referrals, hospitalisations and laboratory results for about 1,500,000 patients from 600 GPs, which is almost 9 % of the total Dutch population. For coding, the Dutch standard for GPs for the classification of symptoms and diseases, the International Classification of Primary Care (ICPC) is used in addition to free text.

### Study population

Data from all individuals between 1 January 2007 and 31 December 2010 in the IPCI database were used for case selection. Date of entry in the study was defined as 1 January 2008 or the date at which 1 year of valid data history in the database was accumulated if this was later. The date of the end of the study was 31 December 2010 or the end of registration of the patient, death or last IPCI data delivery if this was earlier. This entire study population was used to compare age- and gender-specific incidences of migraine before and after the introduction of the HPV vaccination programme. Additionally, a small cohort from this study population was used to compare incidences of migraine in vaccinated and unvaccinated girls and for the SCCS analysis. This cohort consisted of girls born in 1993–1997 from GPs who agreed to make data available for linkage with the vaccination registry (*n* = 20 GPs).

### Case selection

**Fig. 1:**
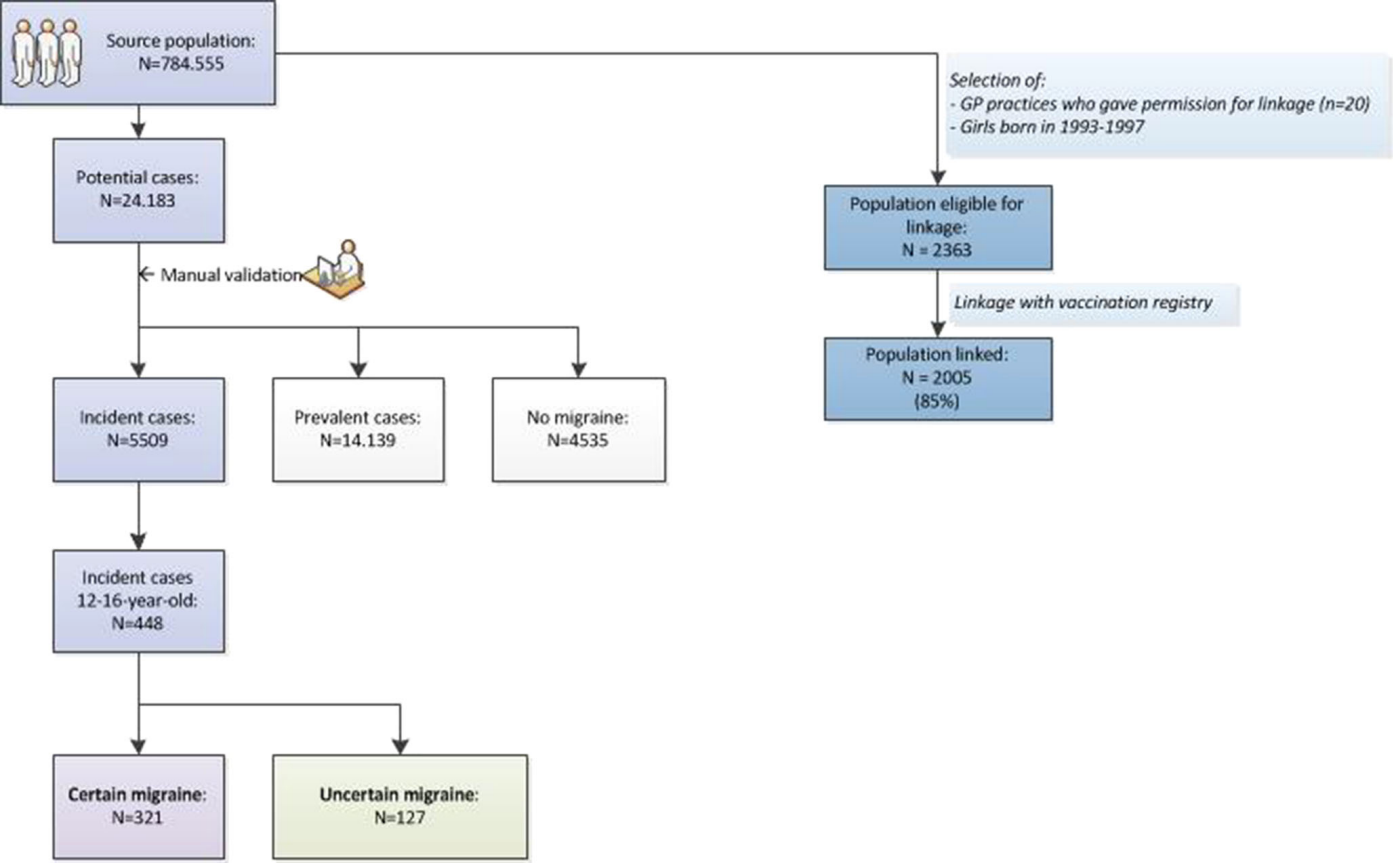
Process of identification of migraine cases

Patients with newly diagnosed migraine were retrospectively identified within the study population. Potential cases were selected if the patient record contained the ICPC-code N89 (migraine) or ‘migrai*’ in the free text during the study period (*n* = 24,183). All potential cases were manually validated by review of the anonymous medical record. Migraine was coded according to the diagnoses of the GP as definite, unclear/possible, menstruation related, medication-overuse headache or no migraine (*n* = 4535). The date of onset of migraine symptoms was defined, or if this was unknown, the date of first entry of migraine symptoms in the patient record was used. Based on this, cases were classified as incident if the onset of first migraine symptoms or first entry of migraine symptoms was within the study period (*n* = 5509 of which 448 are 12–16 years of age), or as prevalent if the patient was diagnosed with migraine before the study period or first symptoms of migraine started before the study period (*n* = 14,139). Prevalent cases were excluded from further analysis. In addition, the occurrence of aura was coded as definite, unclear/possible, typical aura without headache or absent. Finally, two categories were classified, namely ‘certain migraine’ and ‘uncertain migraine’ [7]. Certain migraine refers to patients with definite migraine and menstruation-related migraine (*n* = 321 12- to 16-year olds). Uncertain migraine comprised patients with unclear/possible migraine and typical aura without headache (*n* = 127 12- to 16-year olds). The identification process of migraine cases is described in Fig. 1.

### HPV vaccination exposure

In the Netherlands, all girls aged 13–16 years were invited for HPV catch-up vaccination in 2009, and 12-year-old girls were invited from 2010 onwards. HPV vaccination consists of three successive doses of the bivalent HPV-16/18 vaccine (Cervarix®). All three doses have equal content. Vaccination rounds were organised in March/April, April/May and September/October. However, some girls started later or skipped rounds. Girls not fully vaccinated received another opportunity to complete the series in the next year. All administered doses were registered in the national vaccine registry, Praeventis. Praeventis covers the entire Dutch population in the age groups eligible for vaccination and is updated continuously, as described by Van Lier et al. [29].

Girls born between 1993 and 1997 who were part of the IPCI study population and were patients of the 20 GPs who gave permission for data linkage were linked to the national vaccination registry to determine their HPV vaccination status and the number and dates of vaccine administration (*n* = 2363, Fig. 1). Probabilistic techniques were used for the linkage, because we were not able to use a personal identifier. With this technique, (parts of) personal identifiers, i.e. postal code, date of birth, first name, surname and citizen service number, were taken into account to assess the probability that two records from both databases were from the same person. This linkage technique was validated in an earlier study by Kemmeren et al. [16]. A trusted third party was used to exclude identifying variables from the research database.

### Analysis

**Fig. 2:**
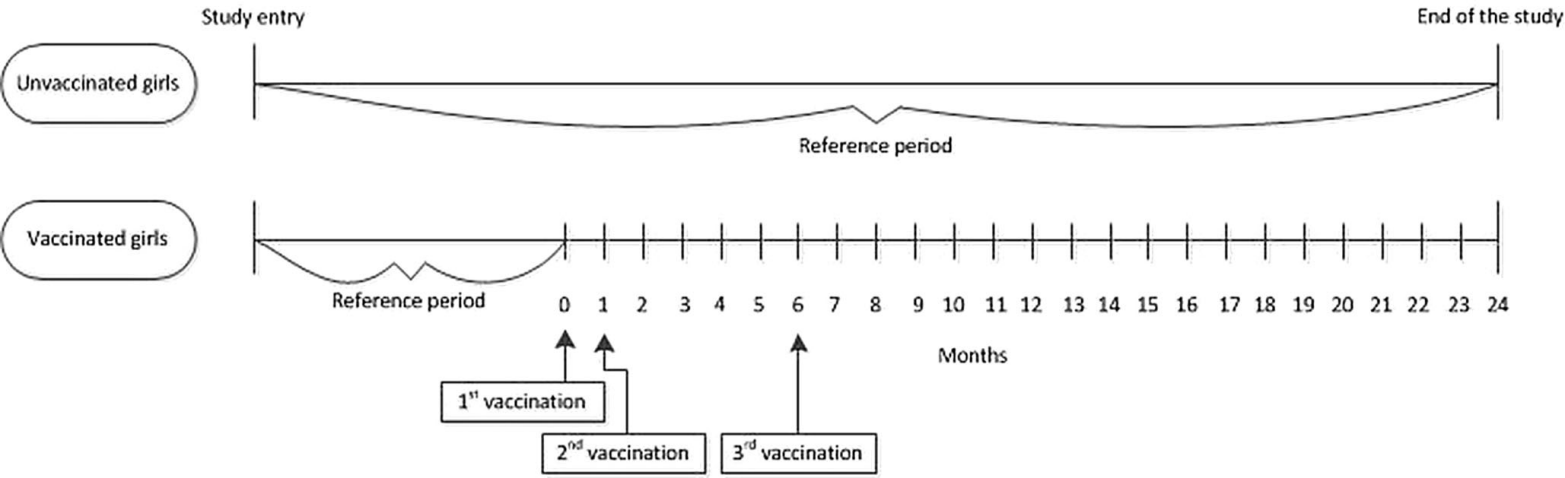
Calculation of the person time used in the cohort analysis

To identify a possible association between HPV vaccination and incident migraine, we analysed the data in three different ways. First, age- and gender-specific incidence rates of migraine were obtained for the period before (pre 2008) and after (post 2009/2010) vaccination. Incidence rate ratios (IRRs) with 95 % confidence intervals were calculated to compare the postvaccination period with the pre-vaccination period. Males are included as a control to indicate trends over time that were independent of HPV vaccination as they were not eligible for HPV vaccination. Second, a cohort analysis was performed to compare monthly incidence rates of migraine between vaccinated and unvaccinated girls. “Unvaccinated” person time comprised the time from entry in the study until the date of the end of the study (see above), the date of occurrence of an event or the date of administration of the first dose, whichever came first. “Vaccinated” person time included the time between administration of the first dose and the date of the end of the study or the date of occurrence of the event, whichever came first (Fig. 2). If the date of the end of the study was earlier than 31 December 2010, the patient was censored. Time after the first dose was divided in months, providing incidence in consecutive months after vaccination. IRRs of these periods compared to the reference period were estimated. Confidence intervals were estimated by the mid-P exact method [20]. Third, SCCS analysis was used to compare the incidence of migraine in the high-risk and non-high-risk periods. Only HPV-vaccinated girls with incident migraine were included in the analysis, whereby each case acts as its own control. A primary high-risk period of 6 weeks following each dose was defined, according to other studies on neurological events following immunisation [6, 10, 24]. Furthermore, three different high-risk periods were defined, one longer period of 13 weeks and two shorter periods of 4 and 2 weeks, to conduct sensitivity analyses. Additionally, we adjusted for school holidays (between 1 July and 31 August and between 20 December and 7 January) because we observed a lower incidence of migraine in those periods. This potentially can cause bias because vaccination was given outside these periods. Furthermore, no seasonality was observed in the incidence of migraine.

## Results

### Pre- and postvaccination period incidences of migraine

The study period comprised a total of 73,245 person years for 12- to 16-year-old boys and girls, in which 321 certain migraine cases and 127 uncertain migraine cases occurred. In 12- to 16-year-old girls, the incidence rate of migraine was slightly higher in the postvaccination period (2009/2010) compared to the pre-vaccination period (2008; Table 1). The IRR for certain migraine was 1.14 (95 % CI 0.82–1.62), and the IRR for certain + uncertain migraine was 1.10 (95 % CI 0.83–1.49). Moreover, in 12- to 16-year-old males, the postvaccination incidence rate of migraine was also slightly higher, but not significantly different, than the pre-vaccination incidence rate (Table 1), with an IRR of 1.21 (95 % CI 0.77–1.97) for certain migraine and 1.20 (95 % CI 0.83–1.78) for certain and uncertain migraine.

**Table 1:** Pre- and postvaccination period incidences of incident migraine for 12- to 16-year-old females and males

	Certain migraine (*n* = 321)	Certain + uncertain migraine (*n* = 448)
Females	Incidence / 100,000 person years (95 % CI)	
2008 (pre-vaccination period)	421 (303–569)	581 (442–752)
2009 (pre-vaccination period)	454 (360–566)	594 (485–721)
2010 (postvaccination period)	505 (408–618)	686 (572–817)
Males	Incidence / 100,000 person years (95 % CI)	
2008 (pre-vaccination period)	208 (130–314)	312 (215–438)
2009 (pre-vaccination period)	234 (170–314)	354 (274–450)
2010 (postvaccination period)	267 (201–349)	394 (312–491)

### Cohort analysis

In the cohort analysis, we included 2005 girls eligible for HPV vaccination in IPCI who could be linked to the vaccination registry. Of these girls, 1306 (65.1 %) received at least one dose of HPV vaccination, 1275 (63.3 %) received at least two doses, and 1228 (61.3 %) received all three doses within the study period. Adherence to the dosing schedule was good: 97 % received the second dose within 2 months after the first, and 84 % of all vaccinated girls received the third dose within 7 months after the first. In 2009/2010, 22 girls had incident migraine (14 certain and eight uncertain), of which 11 (50.0 %) were vaccinated for HPV and 11 (50.0 %) were unvaccinated.

**Fig. 3:**
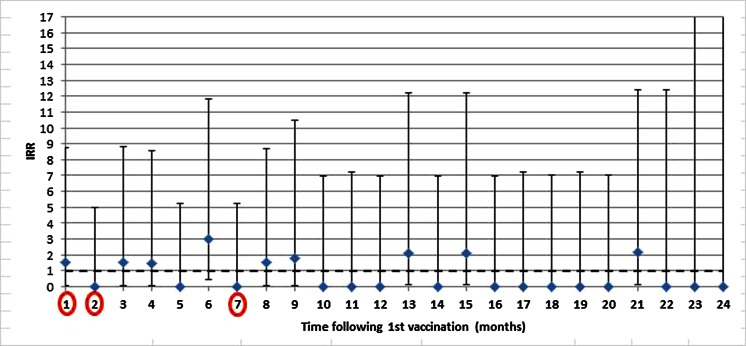
IRRs for incident migraine in monthly periods following the date of first vaccination compared to migraine in unvaccinated girls. Notes: the months following the three doses according to the vaccination schedule are in *red circles*. The *dashed line* represents the cut-off value for an association between HPV vaccination and migraine

**Fig. 4:**
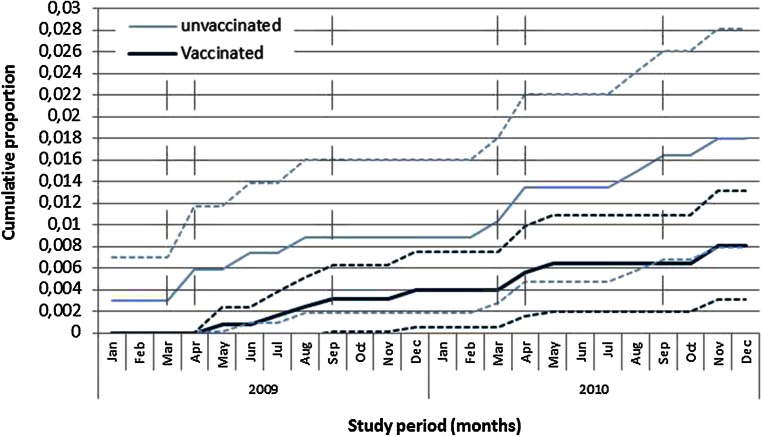
Cumulative proportion of incident migraine with 95 % confidence intervals in HPV-vaccinated and unvaccinated girls during the study period. Note: the *vertical dashed lines* showed the vaccination moments according to the schedule

The IRRs for migraine in monthly periods following the first dose compared to migraine in unvaccinated girls or migraine occurring in the period before vaccination ranged between 0.0 and 3.0 (month 6; Fig. 3). None of these IRRs were statistically significant, and increases in IRRs were not related to the months in which vaccination occurred. Figure 4 shows a lower cumulative proportion of migraine for HPV-vaccinated girls than for unvaccinated girls; however, confidence intervals were overlapping. Increases in the cumulative proportion of migraine during or following vaccination were observed in both vaccinated and unvaccinated girls.

### Self-controlled case series (SCCS) analysis

The association between HPV vaccination and migraine was tested using a hypothesis-testing SCCS study including 11 incident migraine cases in vaccinated girls. Of them, six were certain migraine cases and five were uncertain cases. Ten out of the 11 cases received three doses of HPV vaccine, and one certain case received only one dose. Of the six certain migraine cases, four occurred in 2009 and two occurred in 2010, and of the five uncertain cases, two occurred in 2009 and three occurred in 2010. The mean duration of observation in the study period was 1.8 years (range 0.7–2.0).

Three events occurred in the high-risk period of 6 weeks post HPV vaccination. The events were equally divided over the three doses, and they took place on day 8, 37 and 23 following the first, second and third dose, respectively. We found no statistically significant elevated risk of migraine in the four defined high-risk periods versus non-high-risk periods; however, the risk estimates ranged between 2.1 and 6.3 (Table 2).

**Table 2:** Results of the self-controlled case series (SCCS) analysis for HPV-vaccinated girls with incident migraine

	Certain migraine	Certain + uncertain migraine
6 weeks high-risk period		
Within high-risk period		
Number of events	2^a^	3^b^
Number of days observed	506	984
Non-high-risk period		
Number of events	4	8
Number of days observed	3389	6076
RR (95 % CI) univariate	4.3 (0.69–26.6)	2.9 (0.71–11.7)
RR (95 % CI) adjusted^c^	6.3 (0.80–49.1)	2.8 (0.66–12.0)
Sensitivity analysis	Certain + uncertain migraine RR (95 % CI) univariate	Certain migraine RR (95 % CI) univariate
13 weeks high-risk period	4.6 (0.74–28.5)	3.4 (0.88–12.8)
4 weeks high-risk period	2.2 (0.24–20.3)	2.3 (0.48–11.4)
2 weeks high-risk period	4.5 (0.49–41.2)	2.1 (0.26–16.7)

^a^One following the first dose and one following the second dose

^b^One following the first dose, one following the second dose and one following the third dose

^c^Adjusted for school holidays

## Discussion

In this study, we studied the association between HPV vaccination and migraine. A slightly, but not significantly, higher incidence of migraine among 12- to 16-year-old girls was found in the postvaccination years compared to the pre-vaccination period. Furthermore, we found no significantly higher risk of migraine in high-risk weeks (defined as primary 6 weeks, and secondary 13 weeks, 4 weeks and 2 weeks) after each HPV dose compared to non-high-risk weeks. However, risk point estimates ranged between 2.2 and 6.3 for certain migraine and between 2.1 and 3.4 for certain and uncertain migraine. Finally, no significantly higher risk of migraine was found in the months following vaccination compared to unvaccinated girls.

The number of migraine reports following HPV vaccination in the Netherlands received through the passive safety surveillance system was with eight cases following almost 800,000 doses higher than that in the UK. In the UK, during the first 2 years following introduction of the bivalent HPV vaccine, 17 reports of migraine out of at least 4.5 million doses of HPV vaccine were received through the Yellow Card Scheme [4]. A possible explanation for the difference in reporting rate can be the difference in age groups targeted for HPV vaccination, including the catch-up campaign. In the Netherlands, girls 12 to 16 years of age were invited as in the UK, girls aged 12 up to 18 years. Furthermore, possible differences in spontaneous reporting between countries may lead to different reporting rates. Finally, the adverse media attention for the safety of the HPV vaccine in the Netherlands could have played a role also. Nevertheless, we think it is important to follow up a possible signal to maintain public faith in the NIP as high as possible. To date, little is known about the occurrence of migraine following HPV vaccination despite the fact that headaches are one of the most reported adverse events [9, 11, 19]. Unfortunately, trials in which adverse events were studied did not specify the characteristics of the headaches. However, the frequency of headaches in the week before vaccination was found to be comparable or higher than that in the week following HPV vaccination [25]. Moreover, it is notable that the incidence of migraine among girls rises during puberty. The addition of pubescent girls in the NIP as a new target group for HPV vaccination could have contributed to sudden reports of migraine through the passive safety surveillance system.

The slightly higher, although not statistically significant, incidence of migraine observed among 12- to 16-year-old girls in the postvaccination years compared to the pre-vaccination year, irrespective of vaccination status, was also found for boys in the same age group. This might indicate that there are other unknown causes for an increase in migraine incidence during this period. No changes were made in headache guidelines for GPs before or during the study period. A possible explanation might be the earlier diagnosis of migraine over the years: the incidence of migraine decreased in 30- to 45-year olds and increased in 10- to 29-year olds (data not shown). However, an association is difficult to detect with this method when the effect of HPV vaccination on the incidence of migraine is small or present only for a small time period after vaccination and because only approximately half of the girls were vaccinated against HPV.

The cohort and self-controlled case series analysis also showed no significantly increased risk of migraine in girls who were vaccinated, although the estimated RRs ranged from 2.1 to 6.3. Unfortunately, the number of cases included in the analysis was small, and therefore, we had little power to detect a potential association. The results may depend on the definition of the risk periods, as we did not have an established hazard function or biological mechanism for a potential association. Consistent with studies on other neurological events (e.g. Guillain-Barré syndrome (GBS)) following immunisation, we defined a primary high-risk period of 6 weeks following each dose [6, 10, 24]. In studies of more insidious diseases, such as autoimmune and neurological diseases, after quadrivalent HPV vaccination, longer risk windows of 180 days were used [1, 5]. We conducted a sensitivity analysis to explore the effect of a potential misclassification: we defined three additional high-risk periods, one longer period of 13 weeks and two shorter periods of 4 and 2 weeks. More or less comparable risk estimates were found by using these longer and shorter high-risk periods, which indicates that the 6-week period may not have led to underestimation.

In addition to statistical analyses that may establish an association, a declarative pathophysiological mechanism is of importance prior to determining potential causality. Although we did not find any plausible pathophysiological mechanism that explains how migraine can be caused by HPV vaccination, vaccination may possibly act as a trigger for migraine. However, according to many experts, the value of migraine triggers is overestimated [14], but often highly valued by patients. In many patients, there is a genetic predisposition for migraine, although this is difficult to prove in a complex polygenic disease such as migraine. It is not known why in some patients with a genetic predisposition to migraine develop symptoms and some do not develop symptoms [22, 23]. However, the SCCS method adjusts for such time-independent factors.

A limitation of the study is misclassification of the outcome: underdiagnosis of migraine may have occurred because we used an observational database from GPs. Patients who did not go to a GP do not appear in the database. Furthermore, we defined the date of onset of migraine symptoms. However, if this was unknown, the date of first entry of migraine symptoms in the patient record was used. This may have led to some misclassification, as patients are only likely to consult their GP if multiple headache attacks have occurred. This misclassification could have led to overdiagnosis if the presumed date was within the high-risk period but the actual date of symptom onset was before the vaccination. On the other hand, misclassification of the onset date could have led to underdiagnosis if the presumed date was classified after the high-risk period but the actual date of onset of symptoms lies within the high-risk period.

This study could also include selection bias. First, in this study, a slightly higher percentage of girls was fully vaccinated (61 %) compared to the national vaccination coverage (49–56 %). Vaccinated persons are more likely to be indigenous Dutch and live in areas with higher socioeconomic status [21]. Secondly, only data of a small number of GPs (*n* = 20) was available for linkage to the vaccination registry. Because availability was based on permission of the GP for data linkage, the introduction of selection bias due to this was unlikely.

Finally, residual confounding may have occurred in the SCCS analysis and in the cohort analysis because there was no information available on other risk factors for migraine, such as oestrogen level [13]; therefore, we were unable to adjust for these other risk factors.

In conclusion, using different methods of analysis, no statistically significant association between HPV vaccination and incident migraine was found. Because the number of cases was limited, these results should be interpreted with caution. Larger studies are warranted to investigate this topic.

## Abbreviations

CI: Confidence interval
GBS: Guillain-Barré syndrome
GP: General practitioner
HPV: Human papilloma virus
ICPC: International Classification of Primary Care
IPCI: Integrated Primary Care Information
IRR: Incidence rate ratio
NIP: National Immunisation Programme
SCCS: Self-controlled case series
